# Baseline clinical and biochemical profiles of type 2 diabetes patients enrolled in a lifestyle management program in India, a cross-sectional study

**DOI:** 10.1038/s41598-025-94694-8

**Published:** 2025-03-25

**Authors:** Pramod Tripathi, Nidhi Kadam, Thejas Kathrikolly, Diptika Tiwari, Anagha Vyawahare, Baby Sharma, Malhar Ganla, Banshi Saboo

**Affiliations:** 1 Department of Research, Freedom from Diabetes Research Foundation, ‘Parth’, Ghodke Chowk, Prabhat Road, Pune, Maharashtra 411004 India; 2 Freedom from Diabetes Clinic, ‘Parth’, Ghodke Chowk, Prabhat Road, Pune, Maharashtra 411004 India; 3 Dia Care - Diabetes Hormone Clinic, Gandhi Park, 1 & 2, Nehru Nagar Cir, Opp. BRTS, Bemanagar, Ambawadi, Ahmedabad, Gujarat 380015 India

**Keywords:** Type-2 diabetes, Baseline characteristics, Glycemic control, Obesity, Gender, Diseases, Endocrinology, Health care

## Abstract

Assessing the clinical and biochemical profiles of patients with type-2 diabetes (T2D) is vital for understanding the disease characteristics and improving disease management. This study aimed to assess the profiles of adults (> 18 years old) with T2D enrolled in a one-year diabetes management program in Pune, India. This cross-sectional study retrospectively analyzed 18,950 records from the Freedom from Diabetes Clinic, between November 2015 and June 2023. Baseline sociodemographic, biochemical, and anthropometric parameters were extracted from electronic medical records and analyzed using descriptive statistical methods. A gender-wise comparison was performed for the biochemical and anthropometric measurements. Poor glycemic control (glycated hemoglobin [HbA1c] ≥ 7%) was reported by 70.0% of the patients at baseline, despite the majority (80.2%) being on glucose lowering medications. The prevalence of overweight (body mass index [BMI] 23.0–24.9 kg/m^2^) and obesity (BMI ≥ 25 kg/m^2^) was 21.5% and 57.2%, respectively. Hypertension and hypercholesterolemia were reported in 56.8% and 25.7% of the patients, respectively. A significant association was observed between comorbidities such as hypertension, overweight/obesity, and poor glycemic control (*p* < 0.05). Additionally, significant gender-wise differences were observed in the clinical parameters, including homeostatic model assessment of insulin resistance, fasting insulin, and micronutrients (*p* < 0.05). This study highlights the high prevalence of poor glycemic control, overweight/obesity, and comorbidities among T2D patients enrolled in a one-year diabetes management program in India. Gender-specific differences emphasize the need for tailored management approaches. These findings underscore the importance of comprehensive lifestyle interventions to improve glycemic control, address comorbidities, and reduce complication risks in the Indian population with T2D.

## Introduction

Type 2 diabetes (T2D) is a global health concern that is primarily attributed to lifestyle and associated comorbidities. The International Diabetes Federation (IDF) reports that approximately 537 million adults currently have diabetes, with the number expected to rise to 783 million by 2045^1^. India ranks second in its contribution to the global diabetes epidemic, with nearly 7.2 million people affected in 2021^1,2^. The Asian Indian population is at a higher risk due to specific risk factors such as central obesity, a characteristic "thin-fat" phenotype, and a lower median age at diagnosis^3^. Furthermore, India has a considerable burden of T2D-related comorbidities, such as hypertension, dyslipidemia, and obesity, which present additional challenges in the effective management of T2D^4,5^. Glycemic control, a critical aspect of T2D management, is often intertwined with these comorbidities, which complicate patient management^6^. Considering the complexities of T2D management in this population, it is essential to comprehensively assess clinical and biochemical characteristics to improve overall disease outcomes.

Previous large-scale studies in India, including population-based surveys and limited real-world evidence studies, have primarily focused on reporting the glycemic status, associated risk factors, and complications in specific cohorts of patients with T2D. For instance, the ICMR-INDIAB survey, conducted on a representative population across three Indian states on patients with self-reported diabetes, revealed that 70% had poor glycemic control (HbA1c ≥ 7%)^7^, highlighting a significant gap in diabetes management in the region. Other studies providing real-world evidence have either focused specifically on glycemic control in those receiving antidiabetic therapy in routine healthcare settings^8^ or on younger adults (18–45 years) with diabetes^5^.

The present study, in the context of limited real-world evidence, aimed to undertake an extensive evaluation of the fundamental characteristics of individuals diagnosed with T2D, aged between 18 and 70 years who participated in a one-year intensive lifestyle intervention (ILI)-based diabetes management program from across 560 cities in India. By focusing on this specific cohort, which exhibits significant phenotypic and genotypic differences from Western populations, this study sought to provide a nuanced perspective on the factors influencing glycemic control in the context of structured diabetes management initiatives.

## Methods

### Study design and setting

This was a cross-sectional study of adult (> 18 years old) T2D patients who were part of a one-year in-person (November 2015–January 2020) or a one-year online (February 2020–June 2023) integrated ILI (program for diabetes management in Pune, India. Retrospective data on the baseline anthropometric and biochemical parameters of patients who met the eligibility criteria were extracted from an electronic database maintained by the Freedom from Diabetes Clinic in India.

### Eligibility criteria

The inclusion criteria were age between 18 and 70 years and a confirmed diagnosis of T2D according to the American Diabetes Association (ADA) guidelines^9^. Exclusion criteria were pancreatitis or drug-induced diabetes (steroids), other types of diabetes (type 1 diabetes mellitus, diabetes insipidus, maturity-onset diabetes of the young, latent autoimmune diabetes in adults, or Gestational Diabetes), and myocardial infarction within the last 6 months.

### Data collection

Baseline data of eligible 18,950 patients were retrospectively extracted on the following parameters—

- Sociodemographic data included age, gender, education, and occupation.
- Medical history included the duration of diabetes, comorbid conditions (e.g., cardiovascular disease, hypertension, dyslipidemia, obesity, and kidney function), and medication history. Clinical parameters included hemoglobin, liver, renal, and thyroid function tests, micronutrient status (vitamins D and B12), and serum iron levels.
- Glycemic control included fasting and postprandial blood glucose, HbA1c, and fasting insulin levels. Homeostatic Model Assessment of Insulin Resistance (HOMA-IR) and Homeostatic Model Assessment of Beta-cell function (HOMA-B) were calculated using standard formulas^10^. Patients on external insulin were excluded from the HOMA-IR and HOMA-B analyses.

### Ethical approval

This study adhered to ethical guidelines and all procedures involving human participants were conducted in accordance with the Declaration of Helsinki. Ethical approval for this study was received from the Institutional Ethics Committee of Freedom from Diabetes Research Foundation (Ref. No. FFDRF /IEC/2024/7). This study is registered in the Clinical Trials Registry of India (CTRI) (CTRI/2024/03/064596). The retrospectively extracted data were anonymized and handled with strict confidentiality to ensure the privacy of individuals involved in the study. Personal identifiers were removed to prevent identification of individual participants.

### Patient consent

Due to the retrospective nature of the study, the requirement for informed consent was waived by the Ethics Committee.

### Statistical analyses

Data were analyzed using descriptive statistics including frequency and percentage. Mean ± standard deviation and median (interquartile range) were used to describe normally distributed and skewed data, respectively. The association between categorical variables was assessed using the chi-square test. All analyses were performed using SPSS version 21. Statistical significance was set at *p* < 0.05.

## Results

The mean age of the study population (n = 18,950) was 52.7 ± 10.6 years, and the majority were male (64.6%). The sociodemographic characteristics of the study population are presented in Table 1*.* The study included patients from 560 cities in India.

**Table 1:** Sociodemographic characteristics of the study population.

Parameter	n (%)
Age (years); n = 18,950	
≤ 50	8267 (43.6)
> 50	10,683 (56.4)
Gender; n = 18,950	
Male	12,233 (64.6)
Female	6717 (35.4)
Education; n = 18,656	
Up to 12 years of schooling	1198 (6.4)
Graduate	8449 (45.3)
Post-graduate and above	8337 (44.7)
Preferred not to share	672 (3.6)
Occupation; n = 18,520	
Employed	13,555 (73.2)
Homemaker	2985 (16.1)
Retired	1941 (10.5)
Others (preferred not to share)	39 (0.2)
Family history of diabetes; n = 18,646	
Yes	14,784 (79.3)
No	3862 (20.7)
Dietary Pattern; n = 18,664	
Vegan	2363 (12.7)
Vegetarian	8339 (44.7)
Non-vegetarian	7962 (42.7)

Data are presented as frequencies and percentages.

### Glycemic control and medication status

Glycemic parameters showed that the mean HbA1c level was 8.1 ± 1.8% among the study population, which suggests poor glycemic control despite the majority (80.2%) being on glucose-lowering medication (Oral Hypoglycemic Agents (OHAs) and/or insulin) (Table 2). The median fasting and postprandial blood glucose levels were 131 (110–162) mg/dl and 160 (127–214) mg/dl, respectively. The median fasting insulin levels, HOMA-IR, and HOMA-B were 7.9 (5.0–12.4) µU/mL, 2.7 (1.6–4.4), and 42.6 (23.6–75.5), respectively. Insulin resistance, as measured by HOMA-IR (≥ 2.5)^11^was observed in more than half (54.2%) of the population. A significant difference was observed in the gender-wise comparison of fasting insulin and HOMA-IR with higher levels observed in females than males (*p* < 0.001).

**Table 2:** Glycemic parameters and medication status in the study populations.

Parameter	Total	Males	Females	*p* value	OR (CI)*
HbA1c (%); n = 18,950					
< 7	5681 (30.0)	3697 (30.2)	1984 (29.5)	0.33	1.01 (0.99–1.04)
≥ 7	13,269 (70.0)	8536 (69.8)	4733 (70.5)		
Fasting Blood Glucose (mg/dl); n = 18,182					
70–100	2433 (13.4)	1619 (13.9)	814 (12.5)	0.01	1.04 (1.01–1.07)
> 100	15,749 (86.6)	10,068 (86.1)	5681 (87.5)		
Postprandial Blood Glucose (mg/dl); n = 12,610					
< 140	4306 (34.1)	2723 (33.9)	1583 (34.6)	0.46	0.99 (0.96–1.02)
≥ 140	8304 (65.9)	5307 (66.1)	2997 (65.4)		
Fasting Insulin (mIU/L); n = 15,974					
< 25^12^	15,184 (95.1)	9817 (95.5)	5367 (94.1)	< 0.001	1.14 (1.01–1.20)
≥ 25	790 (4.9)	455 (4.4)	335 (5.9)		
HOMA-IR; n = 15,605					
< 2.5^11^	7150 (45.8)	4895 (48.9)	2255 (40.3)	< 0.001	1.13 (1.11–1.16)
≥ 2.5	8455 (54.2)	5111 (51.1)	3344 (59.7)		
Medication Status; n = 18,950					
On OHA	12,071 (63.7)	7746 (63.3)	4325 (64.4)	< 0.001	
On insulin	746 (3.9)	478 (3.9)	268 (4.0)		
Both OHA and insulin	2379 (12.6)	1404 (11.5)	975 (14.5)		
Drug Naïve	3754 (19.8)	2605 (21.3)	1149 (17.1)		

The data are presented as frequencies and percentages; OHA, oral hypoglycemic agents; HOMA-IR, Homeostatic Model Assessment of Insulin Resistance; HbA1c, glycated hemoglobin; references^11,12^ for corresponding parameter cutoff values; *Reference—female.

### Comorbidities

The prevalence of comorbidities showed that more than half of the study population (57.2%) was obese (BMI ≥ 25 kg/m^2^) with significantly higher percentage of obesity seen in females than males (*p* < 0.001) (Table 3)*.* The prevalence of hypertension (blood pressure ≥ 140/90 mmHg without medication or on anti-hypertensive medication) in the population was 56.8% (42% were on medication). Dyslipidemia, defined as high cholesterol (> 200 mg/dl), elevated triglyceride levels (> 150 mg/dl) and/or high low-density lipoprotein cholesterol (LDL-C) levels (> 100 mg/dl), was also notable. Irrespective of statin therapy status, hypercholesterolemia, elevated triglycerides, and elevated LDL-C was observed in 25.7%, 38.5%, and 53.4% of the population, respectively; 45.3% of participants with dyslipidemia were on statin therapy. The mean LDL-C, total cholesterol, triglyceride, and high-density lipoprotein cholesterol (HDL-C) levels were 104.2 ± 38.3 mg/dl, 172.1 ± 45.6 mg/dl, 153.7 ± 97.9 mg/dl, and 42.1 ± 9.9 mg/dl, respectively. Low HDL-C levels were observed in 57.9% of the population. The mean HDL-C levels were 40.2 ± 9.0 mg/dl (males) and 45.6 ± 10.5 mg/dl (females). Mean triglyceride levels between males and females were comparable; (156.1 ± 105.2 mg/dl vs. 149.4 ± 83.1 mg/dl). Females were significantly more obese than males [BMI 29 (26.8–31.6) kg/m^2^ vs. 27.5 (26.1–29.3) kg/m^2^; (*p* < 0.001)]. Medication status for comorbidities is shown in Table 3.

**Table 3:** Comorbidities in the study population irrespective of medication status.

Parameter	Total	Males	Females	*p* value	OR (CI)^**^
Lipid Profile					
Triglycerides (mg/dl); n = 18,788					
Normal (< 150)	11,562 (61.5)	7418 (61.2)	4144 (62.2)	0.16	0.99 (0.96–1.00)
High (≥ 150)^13^	7226 (38.5)	4708 (38.8)	2518 (37.8)		
LDL-C (mg/dl); n = 18,754					
Normal (< 100)	8736 (46.6)	5881 (48.6)	2855 (42.9)	< 0.001	1.01 (1.06–1.11)
High (≥ 100)^13^	10,018 (53.4)	6219 (51.4)	3799 (57.1)		
Total Cholesterol (mg/dl); n = 10,776					
Normal (< 200)	7902 (73.3)	4707 (75.2)	3195 (70.7)	< 0.001	1.13 (1.10–1.16)
High ((≥ 200)	2874 (26.7)	1549 (24.8)	1325 (29.3)		
HDL-C (mg/dl); n = 18,794*					
Normal	7898 (42.0)	5868 (48.4)	2030 (30.5)	< 0.001	0.77 (0.76–0.79)
Low^13^	10,896 (57.9)	6262 (51.6)	4634 (69.5)		
BMI (kg/m^2^); n = 18,950					
Underweight (< 18.5)	174 (0.9)	113 (0.9)	61 (0.9)	< 0.001	1.18 (1.15–1.21) (normal weight compared with overweight/obese)
Normal (18.5–22.9)^14^	3864 (20.4)	2836 (23.2)	1028 (15.3)		
Overweight (23.0–24.9)	4073 (21.5)	2989 (24.4)	1084 (16.1)		
Obese (≥ 25.0)	10,839 (57.2)	6295 (51.5)	4544 (67.6)		
BP (mmHg); n = 15,326					
Normal (< 140/90)	11,528 (75.2)	7554 (75.2)	3974 (75.3)	0.92	1.0 (0.97–1.03)
High (≥ 140/90)^15^	3798 (24.8)	2492 (24.8)	1306 (24.7)		
TSH (µIU/ml); n = 18,462					
Euthyroid (0.3–5.5)^16^	16,408 (88.9)	10,738 (90.4)	5670 (86.1)	< 0.001	
Hypothyroid (> 5.5)	1775 (9.6)	1013 (8.5)	762 (11.6)		
Hyperthyroid (0.01–0.3)	279 (1.5)	122 (1.0)	157 (2.4)		
Medication Status; n = 18,950					
On anti-hypertensives	7626 (40.2)	4866 (39.8)	2760 (41.1)	0.08	
On statins	8583 (45.3)	5610 (45.9)	2973 (44.3)	0.03	
On cardiac medication	1517 (8.0)	1138 (9.3)	379 (5.6)	< 0.001	
On thyroid supplements	2993 (15.8)	1209 (9.9)	1784 (26.6)	< 0.001	

Data are presented as frequencies and percentages; LDL-C, Low-Density Lipoprotein Cholesterol; HDL-C, High-Density Lipoprotein Cholesterol; BMI, Body Mass Index; BP, Blood Pressure; TSH, Thyroid Stimulation Hormone; *reference range: male, ≥ 40 mg/dl (normal), < 40 mg/dl (low); female, ≥ 50 mg/dl (normal), < 50 mg/dl (low); references^13–16^ for corresponding parameter cut-off values.

^**^Reference—female.

### Other clinical parameters

Nearly 30% of females in the study population had anemia (hemoglobin < 12 g/dl) (Table 4). Additionally, nearly half (46.2%) of the population was Vitamin D deficient, and males were significantly more deficient in micronutrients than females (*p* < 0.001). Elevated high-sensitivity C-reactive protein (hs-CRP) levels were observed in 72.8% of the population, indicating a moderate to high cardiovascular risk. Data on liver, renal, and thyroid functions, along with serum iron and C-reactive protein (CRP) levels, have also been reported; microalbuminuria was observed in more than one-fifth of the population (21.6%). Significant gender-wise differences were observed for total bilirubin, serum albumin, serum creatinine, uric acid, urine microalbumin, thyroid function, and inflammatory markers, namely CRP and hs-CRP (*p* < 0.001).

**Table 4:** Other clinical parameters in the study population.

Parameter	Total n (%)	Descriptives^!^	Male n (%)	Female n (%)	*p* value	OR (CI)^o^
Hemoglobin (g/dl)						
n = 18,656*						
Normal	12,598 (67.5)	13.8 ± 2.6	7999 (66.4)	4599 (69.5)	< 0.001	1.05 (1.03–1.08)
Deficient^17^	6058 (32.5)		4044 (33.6)	2014 (30.5)		
Micronutrients						
Vitamin B12 (pg/ml); n = 17,993						
Normal (≥ 200.0)	14,095 (78.3)	305 (211–482)	8792 (75.6)	5303 (83.2)	< 0.001	1.16 (1.14–1.19)
Deficient (< 200.0)^18^	3898 (21.7)		2831 (24.4)	1067 (16.8)		
Vitamin D (ng/ml); n = 18,131						
Normal (≥ 20.0)	9757 (53.8)	21.2 (13.7–31.9)	5920 (50.7)	3837 (59.4)	< 0.001	1.13 (1.11–1.16)
Deficient(< 20.0)^19^	8374(46.2)		5746 (49.3)	2628 (40.6)		
Liver function						
Total Bilirubin (mg/dl); n = 18,457						
Normal (0.1–1.3)	17,638 (95.6)	0.7 ± 1.8	11,141 (93.6)	6497 (99.1)	< 0.001	0.68 (0.66–0.69)
Hyperbiluribinemia (> 1.3)^20^	819 (4.4)		762 (6.4)	57 (0.9)		
Total Protein (gm/dl); n = 18,165						
Normal (6.0–8.0)	17,402 (95.8)	7.3 ± 5.8	11,240 (95.9)	6162 (95.7)	0.54	1.02 (0.96–1.08)
High (> 8.0)^21^	763 (4.2)		484 (4.1)	279 (4.3)		
Serum Albumin (gm/dl); n = 17,957						
Normal (3.5–5.0)	17,591 (98.0)	4.4 ± 3.1	11,304 (97.3)	6287 (99.3)	< 0.001	0.74 (0.71–0.77)
High (> 5.0)^21^	366 ( 2.0)		319 (2.7)	47 (0.7)		
Renal function						
eGFR (ml/min/1.73 m^2^); n = 17,072						
Normal (≥ 60.0)	16,440 (96.3)	100.4 ± 21.5	10,666 (96.4)	5774 (96.1)	0.41	0.98 (0.92–1.04)
Low (< 60.0)^22^	632 (3.7)		400 (3.6)	232 (3.9)		
Urine Microalbumin (µg/min); n = 14,184						
Normal (< 20.0)	11,117 (78.4)	7.2 (3.9–17.9)	7000 (77.0)	4117 (80.9)	< 0.001	0.92 (0.90–0.95)
Microalbuminuria (20.0–200.0)^23^	3067 (21.6)		2094 (23.0)	973 (19.1)		
Serum Creatinine (mg/dl)^‡^						
n = 18,868						
Normal	17,553 (93.0)	0.81 ± 0.39	11,151 (91.5)	6402 (95.9)	< 0.001	0.80 (0.78–0.83)
High^23^	1315 (7.0)		1039 (8.5)	276 (4.1)		
Uric Acid (mg/dl)^#^						
n = 16,167						
Normal	14,577 (90.2)	5.3 (4.4–6.2)	9453 (88.6)	5124 (93.2)	< 0.001	2.21 (1.95–2.50)
High^24^	1590 (9.8)		1217 (11.4)	373 (6.8)		
Blood Urea Nitrogen (mg/dl); n = 16,097						
Normal (5.0–20.0)	15,663 (97.3)	10.9 ± 7.9	10,105 (97.2)	5558 (97.5)	0.37	0.97 (0.91–1.04)
High (> 20.0)^25^	434 (2.7)		289 (2.8)	145 (2.5)		
T3 (ng/dl); n = 16,165						
Normal (80.0–180.0)	13,110 (81.1)	99 (85–113)	8602 (82.2)	4508 (79.1)	< 0.001	0.94 (0.78–1.12)
High (> 180.0)^26^	50 (0.3)		35 (0.3)	15 (0.3)		
Low (< 80.0)	3005 (18.6)		1828 (17.5)	1177 (20.6)		0.93 (0.90–0.96)
T4 (µg/dl); n = 16,196						
Normal (5.6–12.0)	14,656 (90.5)	8.5 (7.3–9.7)	9561 (91.2)	5095 (89.3)	< 0.001	
High (> 12.0)^26^	694 (4.3)		294 (2.8)	400 (7.0)		1.54 (1.41–1.68)
Low (< 5.6)	846 (5.2)		633 (6.0)	213 (3.7)		1.15 (1.10–1.20)
Additional parameter						
Iron (μg/dl)^**^; n = 17,423						
Normal (55.0–175.0)	14,684 (84.2)	79 (61.0–99.9)	10,050 (89.5)	4634 (74.8)	< 0.001	
High (> 175.0)^27^	104 (0.6)		92 (0.8)	12 (0.2)		0.77 (0.72–0.83)
Low (< 55.0)	2635 (15.1)		1084 (9.7)	1551 (25.0)		1.67 (1.59–1.74)
CRP (mg/dl); n = 3936						
Normal (< 0.3)	195 (5.0)	2.7 (1.0–6.2)	134 (5.7)	61 (3.9)	0.01	1.16 (1.05–1.28)
High (≥ 0.3)^28^	3741 (95.0)		2223 (94.3)	1518 (96.1)		
HsCRP; n = 15,494						
Low CV risk (< 1.0)	4221 (27.2)	2.0 (0.9–4.2)	3272 (32.8)	949 (17.2)	< 0.001	
Moderate CV risk (1.0–3.0)	5805 (37.5)		3995 (40.0)	1810 (32.9)		1.13 (1.10–1.15)
High CV risk (> 3.0)^29^	5468 (35.3)		2722 (27.2)	2746 (49.9)		1.56 (1.51–1.61)

Data are presented as frequencies and percentages; Descriptive statistics are presented as mean ± standard deviation (SD) for normally distributed variables or median (interquartile range) for skewed variables; eGFR, estimated glomerular filtration rate; TSH, thyroid-stimulating hormone; CRP, C-reactive protein; hsCRP, high-sensitivity C-Reactive protein; *reference range: male – ≥ 14 g/dl (normal), < 14 g/dl (low); female—≥ 12 g/dl (normal), < 12 g/dl (low)

^‡^ reference range: male – < 1.12 mg/dl (normal), ≥ 1.12 mg/dl (high); female—< 1.01 mg/dl (normal), ≥ 1.01 mg/dl (high); ^#^reference range: male—3.5–7.2 mg/dl (normal), > 7.2 mg/dl (high); female–pre-menopause- 2.6–6.0 mg/dl (normal), > 6.0 mg/dl (high), post menopause—0.5–7.2 mg/dl (normal), > 7.2 mg/dl (high)

**Reference range: male—55.0–175 μg/dl (normal), > 175 μg/dl (high), < 55 μg/dl (low); female—50.0–170.0 μg/dl (normal), > 170 μg/dl (high), < 50.0 μg/dl (low); references^17–29^ for corresponding parameter cut-off values.

^o^Reference–female.

### Association between comorbidities and glycemic control

We observed a significant association between comorbid conditions, such as hypertension, overweight/obesity, and poor glycemic control (HbA1c ≥ 7%) (Table 5)*.* Comorbidities such as overweight/obesity (BMI ≥ 23.0 kg/m^2)^ and hypertension (blood pressure ≥ 140/90 mmHg) were significantly associated with poor glycemic control (HbA1c ≥ 7%). Additionally, more than six years of diabetes duration demonstrated a significant positive association with poor glycemic control (*p* < 0.05).

**Table 5:** Association of clinical and anthropometric characteristics with glycemic control (HbA1c).

Parameters	HbA1c < 7% N (%)	HbA1c ≥ 7% N (%)	*p* value
Age (years)			
≤ 50	2526 (44.5)	5741 (43.3)	0.13
> 50	3155 (55.5)	7528 (56.7)	
BMI (kg/m^2^)			
Normal (18.5–22.9)	1364 (24.2)	2500 (19.0)	< 0.001
Overweight/Obese (≥ 23.0)	4262 (75.8)	10,650 (81.0)	
BP (mmHg)			
Normal (< 140/90)	3687 (79.1)	7841 (73.5)	< 0.001
High (≥ 140/90)	973 (20.9)	2825 (26.5)	
Disease duration (years)			
< 6	1945 (34.2)	2897 (21.8)	< 0.001
≥ 6	3736 (65.8)	10,372 (78.2)	

Data are presented as frequency and percentage. BMI, Body Mass Index; BP, Blood Pressure; HbA1c, Glycated hemoglobin.

## Discussion

This cross-sectional study provides a comprehensive analysis of the baseline characteristics and glycemic challenges of 18,950 patients diagnosed with T2D who participated in an intensive lifestyle intervention program for diabetes management in India. The prevalence of poor glycemic control among most of the study population, coupled with significant associations between suboptimal glycemic control and comorbidities, such as hypertension, dyslipidemia, and obesity, underscores the need for effective and targeted interventions in the management of T2D.

We report the prevalence of poor glycemic control, as assessed by HbA1c levels (≥ 7%), in a substantial proportion of patients (70%) despite the use of glucose-lowering medications. This finding is consistent with the results of the multicenter ICMR-INDIAB study, which reported poor glycemic control in 69% of the population^7^. Similarly, another large-scale cross-sectional study of 55,639 individuals showed that 76.6% of the population had uncontrolled T2D^8^.The reason for this suboptimal glycemic control could be attributed to previous reports of increased non-adherence to pharmacotherapy management in the Asian population^30^, or it could highlight the inadequacy of pharmacotherapy as a standalone management tool for T2D, especially in the Asian/Asian-Indian population.

Our study highlights the significant burden of T2D and comorbidities, with nearly half of participants being overweight or obese, 56.8% showing hypertension, 26–53% showing dyslipidemia, and 70% having T2D for over 6 years, emphasizing the need for tailored interventions. Comorbidities such as hypertension, dyslipidemia, and cardiovascular disease increase the risk of diabetes-related complications, posing challenges in management and secondary prevention while also impacting the quality of care for patients with T2D^31,32^. Further, we found that poor glycemic control was significantly associated with elevated blood pressure (which is a leading cause of cardiovascular-related and premature deaths worldwide, and an independent risk factor for macrovascular and microvascular complications)^33^, higher BMI (> 23.0 kg/m^2^), and longer disease duration (> 6 years), consistent with previous studies^34,35^. These findings underscore the importance of integrated management strategies that address lifestyle factors and early intervention to improve glycemic control and reduce complications.

Gender-wise comparison of comorbidities in our study found higher levels of cardiometabolic risk factors, including dyslipidemia (high LDL-C, high total cholesterol with low HDL-C), obesity and hypothyroidism among women, whereas men showed higher cardiac medication use, reflecting distinct disease patterns^36^. Our findings on gender-specific lipid profile align with previous studies that report high LDL-C and triglyceride levels among women, contributing to a substantial cardiovascular risk among them^37,38^. Nevertheless, few research on lipid profile differences in T2D populations have reported that men with T2D tend to have high triglyceride levels, predisposing them to cardiovascular risk especially at younger ages^37,39^. Further, studies also indicate that while men with diabetes have a 3.8-fold increased risk of coronary heart disease (CHD), women face a significantly higher 14.7-fold risk, even after adjusting for traditional factors^40^. Women may be more vulnerable to cardiovascular disease (CVD) due to multiple factors, including the loss of estrogen’s protective effects post-menopause, which contributes to a more atherogenic lipid profile^41^. Additionally, women often exhibit worse cardiometabolic profiles than men, with higher visceral adiposity, hypertension, and dyslipidemia, further increasing CVD risk^42^. These results highlight the need to consider gender-specific risk factors when addressing CVD risk in patients with diabetes.

Our study further highlights gender-specific differences in other clinical parameters. Consistent with previous research, females had higher fasting insulin levels and HOMA-IR, likely due to greater visceral fat, contributing to metabolic dysfunction and cardiovascular risk^43–45^. Additionally, men showed more vitamin B12 and D deficiencies, while women had higher thyroid dysfunction (elevated T3, T4, and TSH levels) and lower iron levels, likely due to hormonal, dietary, and physiological factors^46,47^.

Despite a higher prevalence of obesity, women in our study maintained higher vitamin D levels than men, which can be attributed to multiple physiological and behavioral factors. Increased use of vitamin D supplements and awareness of its sources may contribute to better vitamin D status despite increased adiposity^48^. Estrogen enhances vitamin D metabolism by increasing serum levels of its active form, potentially mitigating the effects of obesity on vitamin D levels^49^. Additionally, women have a higher proportion of subcutaneous fat, which may store and release vitamin D more effectively than visceral fat, which is more common in men and associated with lower vitamin D levels^50^. Lifestyle factors such as sun exposure and higher consumption of dairy or fortified foods among women may also contribute to these findings^51^.

Overall, such gender-specific associations with various clinical parameters highlight the complexity of gender differences and emphasize the need for tailored approaches for effective T2D management. Considering the multifactorial aspects of diabetes management, healthcare professionals must consider lifestyle, culture, socioeconomic status, and medication regimens when providing personalized care^8^. By using targeted interventions such as patient education, lifestyle counselling, and tailored medication management, healthcare providers can help improve glycemic control and reduce the clinical burden of comorbidities in patients with T2D.

## Limitations

This cross-sectional study provides valuable insights into the baseline characteristics of a large cohort of patients with T2D in India. However, it has certain limitations. As a retrospective analysis of data from a clinical database, this study was susceptible to biases related to data completeness. The cross-sectional design enabled the identification of associations between variables, but did not establish causation or track disease progression and treatment response over time. Additionally, the single-center design may have introduced a selection bias. Although this could have limited the generalizability of the study to the broader Indian population, the hybrid nature of the program helped mitigate this to some extent by enrolling patients from 560 cities across India. The subscription-based model may also have introduced socioeconomic selection bias, restricting participation to those who could afford it and potentially underrepresenting lower-income groups, which may have impacted the findings. Furthermore, this study did not assess the history of self-reported complications, which could have influenced the overall assessment.

## Conclusion

Despite these limitations, our study on Indian population from across 560 Indian cities provides a comprehensive assessment of T2D patients enrolled in a diabetes management program. With 70% of patients experiencing poor glycemic control despite treatment, targeted interventions are urgently needed. The strong association between population-specific comorbidities such as hypertension, dyslipidemia, obesity, and suboptimal glycemic control underscores the complexity of T2D management. A holistic approach, considering sociodemographic factors and comorbidities, is essential for effective diabetes care. Future longitudinal studies are needed to explore causative relationships and trends in glycemic control.

## Data Availability

All data are available from the corresponding author and can be shared following a reasonable request. In such cases, patient information will be de-identified before sharing.
